# Differences between Practice Patterns of Conventional and Naturopathic GPs in Germany

**DOI:** 10.1371/journal.pone.0163519

**Published:** 2016-10-03

**Authors:** Gunter Laux, Berthold Musselmann, Marion Kiel, Joachim Szecsenyi, Stefanie Joos

**Affiliations:** University Hospital Heidelberg, Department of General Practice and Health Services Research, Heidelberg, Germany; University of South Australia, AUSTRALIA

## Abstract

**Background:**

Limited evidence exists whether practice patterns of general practitioners (GPs) who have additionally completed training in naturopathy are different from those of conventional GPs. We aimed to assess and compare practice patterns of GPs in conventional and naturopathic GPs.

**Methods:**

Routine data from 41 GPs (31 with and 11 without additional qualification in NP, respectively) and 180,789 patients, drawn from the CONTinuous morbidity registration Epidemiologic NeTwork (CONTENT)-registry and collected between 2009 and 2014, were used. To assess practice patterns determinants of (non-)phytopharmaceutical prescriptions, referrals and hospitalizations were analyzed using mixed-effects Poisson regression models. As explanatory variables, the qualification of the GP in NM, the age group and sex of the patient, as well as bivariate interactions between these variables were considered.

**Results:**

GPs additionally qualified in naturopathy exhibited higher rates of phytopharmaceutical prescriptions (p<0.034; independent effect) compared to conventional GPs. This association was not observed with respect to non-phytopharmaceutical prescriptions. However, interaction effects between qualification and age group as well as sex were present with respect to both phytopharmaceutical and non-phytopharmaceutical prescriptions (all p<0.001). No further independent association existed between qualification and either referral rates or hospitalization rates, but again interactions between qualification and age group and sex (only referrals) were statistically significant (all p<0.0001).

**Conclusion:**

The results show that the rate of phyto-pharmaceutical prescriptions are generally higher when the GP has an additional qualification in naturopathy. Further differences in practice patterns between conventional and naturopathy GPs could be subject to certain age groups and sex. However, the magnitude of these differences seem to be rather small.

## Background

The growing popularity of naturopathic approaches and Complementary and Alternative Medicine (CAM) among patients is associated with an ongoing debate of integrating such therapies into mainstream healthcare. Naturopathy can be classified under the broad term of CAM, often even used synonymously for it in German. CAM as well as the use and provision of CAM differ significantly between countries [1]. Following a salutogenetic approach naturopathic doctors see their role in ‘supporting the body’s ability to maintain and restore health, preferring natural and less invasive treatment approaches’ [2]. In Germany naturopathy is deeply embedded in the society. The pillars of naturopathy—as understood in the German-speaking area—are the five Kneipp therapies: hydrotherapy, exercise, nutrition, phytotherapy (the use of plants or plant extracts) and lifestyle management (= German: Ordnungstherapie) [3].

In some countries there are recognized licensures for naturopathic physicians trained as primary care physicians in 4-year, accredited doctoral-level naturopathic medical schools. At present, there are 15 US states, 2 US territories, and several provinces in Canada, Australia, and New Zealand that recognize licensure for naturopathic physicians [4]. In Germany, all physicians practicing in direct patient care can obtain an additional qualification in naturopathy by the Medical Chamber after a theoretical and practical training (German: “Zusatzqualifikation Naturheilverfahren”).

The duration of the training is typically three months with a physician authorized by the Medical Chamber to provide postgraduate training in naturopathy complemented by 80 hours of case-seminars including supervision and 160 hours attendance of a seminar program. According to the current curriculum physicians acquire knowledge, experience and skills in several areas of naturopathy, including phytotherapy, balneotherapy, massage therapy and manual diagnostics, nutritional medicine, regulative therapy, physical therapy and neural therapy.

At the end of 2014, a number of 16.323 NP qualifications were registered among all 360.000 German physicians actively working in Germany [5]. The vast majority of naturopathy qualifications are distributed among physicians working in the outpatient sector and in particular in general practice. Thus, we can assume that about 15–20% of all GPs in Germany have completed a training in naturopathy in Germany. Moreover, it is known that doctors with an additional qualification in naturopathy more frequently use and recommend CAM therapies such as acupuncture, manual medicine, relaxation techniques. This means that the presence of a naturopathic qualification can be regarded as an indicator for a general attitude and orientation towards CAM.

It is well known that CAM is widely used in Western countries. However, quantification is problematic as shown by a systematic review assessing CAM prevalence across the EU. Most studies are generally poor and heterogeneous [6]. In Germany, in the last 10–15 years more and more doctors in particular GPs have integrated CAM in their everyday practice. Previous studies suggest that the provision of CAM is particularly widespread in the outpatient setting [7]. In a national survey we have shown that about 60% of German GPs use some type of CAM—with or without having a specific additional qualification for this [8]. Interestingly, data of the same survey point in the direction that GPs using CAM have a higher job satisfaction [9].

While we have a huge body of evidence for the use and distribution of CAM studies exploring the role of CAM from the perspective of health care systems are scarce. Studies from the Netherlands come to the conclusion that patients whose GP has an additional qualification in CAM have lower healthcare costs resulting from fewer hospital stays and prescription rates [10, 11]. To differentiate selection effects from "real" differences in quality of care the authors call for more studies exploring to what extent selection effects could explain the lower costs of patients. In a Swiss study the authors describe better cost-effectiveness for CAM with a different cost structure (less costs for medication) and higher patient-reported quality of care in GPs with CAM qualification [12]. A 2012 published systematic review on economic evaluations of complementary therapies and integrative found that higher-quality studies indicate potential cost-effectiveness, and even cost savings across a number of CAM therapies [13].

In the context of CAM the understanding of decision making processes and prescribing behavior in primary care is essential. However, studies investigating prescribing behavior of GPs are scarce. As we know from previous studies assessing decision making regarding antibiotic prescriptions in patients with an acute respiratory tract infection prescribing seems to depend more on the attending doctor's prescribing behaviour and training than on the clinical picture or patient characteristics [14, 15]

Until now, to our knowledge, a study investigating potential differences in practice patterns of GPs with regard to an additional qualification in naturopathy for the German healthcare system is missing. Therefore, the aim of our study was to evaluate and compare practice by GPs with and without an additional qualification in naturopathy. For this reason, we compared the rates of phytopharmaceutical prescriptions, non-phytopharmaceutical prescriptions, pharmaceutical prescriptions in general, referrals and hospitalizations between these two groups of GPs.

## Methods

An observational study using routine data from general practices in the German healthcare system was conducted.

### Data source

The data for this study were drawn from the CONTinuous morbidity registration Epidemiologic NeTwork (CONTENT)-registry. CONTENT is a general practice research network in Germany that has been described in detail elsewhere [16, 17]. In brief, it provides a platform for continuous, episode-based registration of primary care data. As a special feature, it uses the International Classification of Primary Care (ICPC) to record primary care encounters. Currently, CONTENT contains data from more than 200,000 patients and more than 4 million patient encounters. The participating practices are located in different areas (rural, suburban and urban) in four Germany federal states (Baden-Württemberg, Hesse, Lower Saxony and Rhineland-Palatinate). The data used for this study comprise routine claims data from GPs as they are regularly collected in the German healthcare system. The CONTENT study protocol was approved by the ethics committee of the University of Heidelberg (approval number 442/2005).

### Eligibility criteria

The data from all GPs who submitted their routine data to CONTENT over the period from 1 April 2009 to 31 March 2014 were included. The study population comprised 41 GPs, of which 11 (27%) had an additional qualification in NM and 31 (73%) had no such qualification.

### Variables

Outcomes of interest in this study were rates of phytopharmaceutical prescriptions, non-phytopharmaceutical prescriptions, pharmaceutical prescriptions in general, referrals to other physicians and hospitalizations. Special emphasis was given here to phytopharmaceutical prescriptions, since an increased prescription rate by GPs qualified in NM appears likely due to their specific training in phytopharmaceutical therapy.

Each prescribed pharmaceutical preparation can be identified by a unique number within the CONTENT registry. This number can be used to unambiguously determine the code of the Anatomical Therapeutic Chemical (ATC) classification [18]. On the basis of the ATC code, each preparation can be determined in detail up to and including the chemical substances and especially could be used for this study to determine if a prescription was phyto-pharmaceutical or not.

As explanatory variables, we considered the qualification of the GP (with or without additional qualification in NP), as well as patients’ the age group (<20 years, 20–29 years. 30–39 years, …, 70–79 years, >79 years) and patients’ gender.

### Statistical analysis

The data from individual visits to the GP practice were aggregated at the physician-level (for descriptive analyses only) and at the patient-level (for regression analyses). Mixed-effects Poisson regression models were fitted for each outcome (i) with the qualification as the only covariate in the model (unadjusted model), (ii) with age group and sex as additional covariates, and (iii) with all explanatory variables and their bivariate interactions (full model) [19]. A random physician effect was included in these models to take into account the clustered data structure (multiple observations per physician) and the logarithm of the number of visits per patient was included as an offset. Stepwise variable selection was then performed with the full model until only predictors variables remaining in the model were statistically significantly associated with the outcome at the 5%-level, based on Type III tests [19] of fixed effects. To facilitate interpretability of the final model results in the presence of interactions, the models were used to predict the rates of prescriptions, referrals and hospitalizations per 100 visits according to each age group and sex for physicians with and without additional qualification in NM, respectively. Since the study is exploratory, all p values from statistical tests are to be interpreted descriptively.

The analyses were performed using SAS 9.4 (SAS Institute Inc., Cary, North Carolina, USA). PROC GLIMMIX was used for mixed-effects regression analyses. Figures illustrating the predicted rates of the outcomes were prepared in R version 3.2.0 (R Foundation for Statistical Computing, Vienna, Austria).

## Results

### Study population

The 11 GPs with an additional qualification in NM and the 30 GPs without an additional qualification treated 180,789 patients in total. Basic demographic characteristics of these patients according to the qualification of physicians are shown in Table 1. No major differences in the distribution of patients according to age and sex were observed.

**Table 1:** Study population.

Characteristics		GP with qualification in NM	GP without qualification in NM
Patients, n (%)		36,183 (20)	144,606 (80)
Female, n (%)		22,150 (61)	86,209 (60)
Age			
	Mean (SD)	50 (22)	50 (23)
	Median (IQR)	50 (31, 67)	49 (33, 66)

Abbreviation: GP, general practitioner; interquartile range, IQR; NM, naturopathic medicine; SD, standard deviation.

### Basic models

Crude rates of the outcomes as well as relative rates, both unadjusted and with basic adjustment for age group and sex, are shown in Table 2. For all outcomes, except phytopharmaceutic prescriptions, GPs with and without additional an additional qualification in NP exhibited similar rates. The rate of phytopharmaceutic prescriptions was 1.7 per 100 patient visits for GPs not qualified in NP and approximately 50% higher (2.7 per 100 visits) for GPs qualified in NP (p = 0.04; adjusted for age group and sex of patients).

**Table 2:** Crude outcomes.

Outcome		Crude rate per 100 visits		Relative rate			
		GP with qualification in NM	GP without qualification in NM	Unadjusted		Adjusted for age group and sex	
		Est. (95%-CI)	Est. (95%-CI)	Est. (95%-CI)	P value	Est. (95%-CI)	P value
Prescriptions		** **	** **	** **	** **	** **	** **
	Phytopharmaceutic	2.7 (2.0, 2.6)	1.7 (1.5, 2.1)	1.50 (1.06, 2.13)	0,02	1.41 (1.02, 1.95)	0,04
	Non-phytopharmaceutic	96.8 (81.8, 114.5)	89.4 (80.8, 99.0)	1.08 (0.89, 1.32)	0,43	1.11 (0.92, 1.36)	0,28
	Any	99.7 (84.3, 117.8)	91.4 (82.6, 101.1)	1.09 (0.90, 1.33)	0,39	1.12 (0.92, 1.36)	0,25
Referrals		18.9 (16.5, 21.7)	20.0 (18.4, 21.7)	0.94 (0.81, 1.11)	0,49	0.92 (0.79, 1.08)	0,34
Hospitalizations		1.06 (0.65, 1.72)	0.96 (0.72, 1.29)	1.10 (0.62, 1.94)	0,74	1.13 (0.64, 0.69)	0,74

Abbreviation: CI, confidence interval; Est., estimate; GP, general practitioner; NM, naturopathic medicine. P values are based on Type III tests of fixed effects.

### Pharmaceutical prescriptions

**Fig 1:**
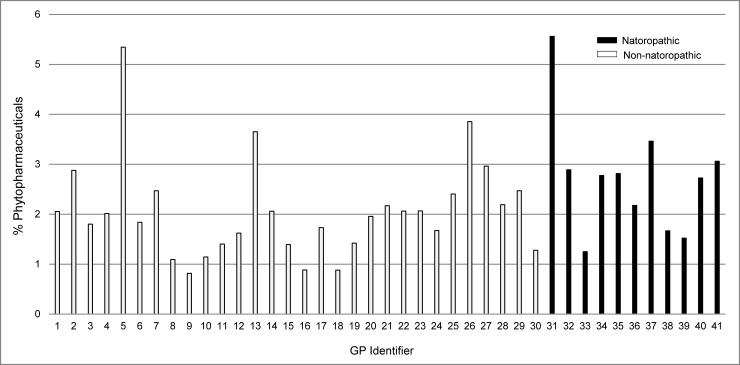
Percentage of phytopharmaceutical prescriptions by physician.

**Fig 2:**
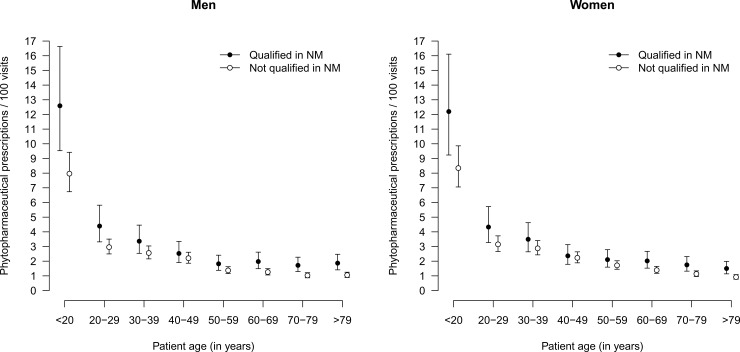
Rates of phytopharmaceutical prescriptions according to qualification of physician, stratified by age group and sex of patients.

**Fig 3:**
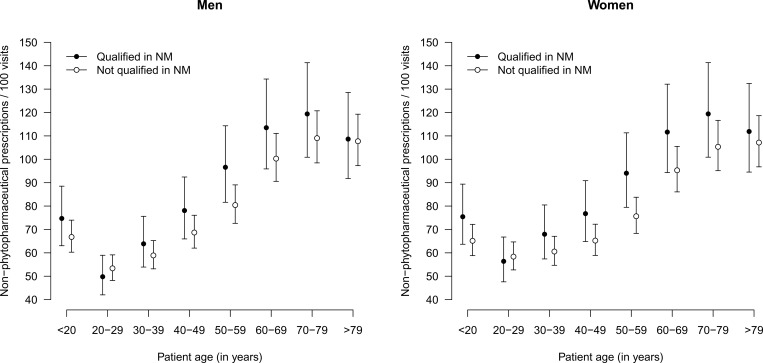
Rates of non-phytopharmaceutical prescriptions according to qualification of physician, stratified by age group and sex of patients.

The percentage of phytopharmaceutical prescriptions of each of the included GPs is depicted in Fig 1. It shows that this value ranges between 1% and 5–6% in both groups of GPs. Model-based predictions of phytopharmaceutical prescriptions according to the qualification of the GP, age group and sex, as depicted in Fig 2, reveal that these prescriptions are more frequent in younger age groups, especially in patients aged younger than 20 years. The qualification of the GP was independently associated with the rate of phytopharmaceutical prescriptions and consistently slightly higher for GPs additionally qualified in NM (p = 0.034). By contrast, the rates of non-phytopharmaceutical prescriptions exhibited a different pattern across the different subgroups of patients, as shown in Fig 3. They were lowest in the age group 20–29 years and increased steadily until the age group 70–79 years. Although the estimates rates were higher with GPs qualified in NM in the age groups from 30 years to 79 years, the qualification was no independent predictor of the rate of non-phytopharmaceutical prescriptions or pharmaceutical prescriptions in general. However, interaction effects between the qualification and age group as well as sex were observed with respect to phytopharmaceutical prescriptions, non-phytopharmaceutical prescriptions and pharmaceutical prescriptions in general (all p<0.001). See S1 Table for the identified determinants of all analyzed types of prescription rates.

### Referrals

**Fig 4:**
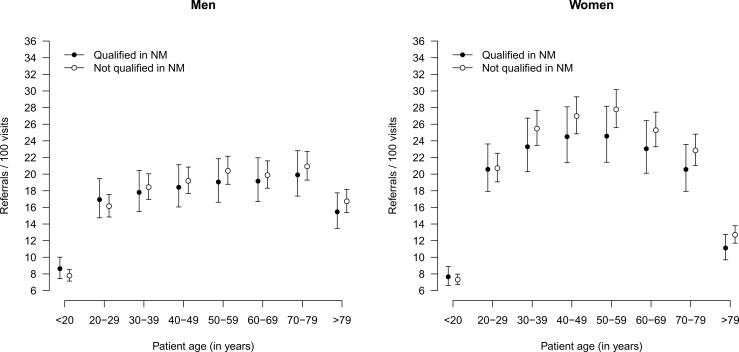
Rates of referrals according to qualification of physician, stratified by age group and sex of patients.

Referral rates were considerably lower for the youngest and the oldest age group compared to the remainder of the age groups with respect to both sexes, as is shown in Fig 4. They also tended to be higher for women. Referral rates by GPs with additional qualification in NM were mostly in the same order of magnitude. Again, interaction effects between the qualification in NP and age group as well as sex were observed with respect to referral rates (both p<0.0001), but referrals were not generally higher or lower given that a qualification in NP was present or not. See S2 Table for the identified determinants of referral rates.

### Hospitalizations

**Fig 5:**
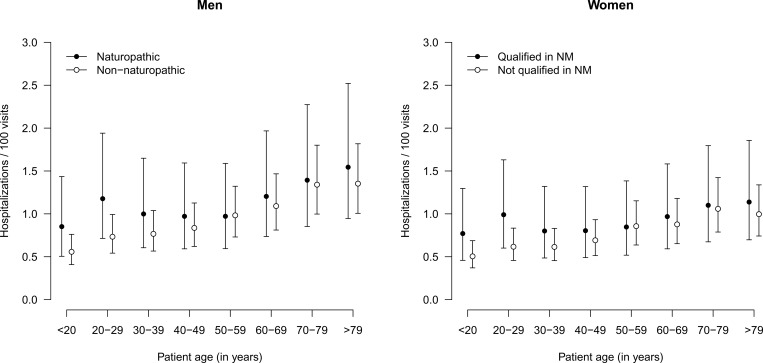
Rates of hospitalizations according to qualification of physician, stratified by age group and sex of patients

In most strata, hospitalizations occurred approximately in 1 per 100 visits, as is depicted in Fig 5. A slight tendency towards higher hospitalization rates in younger patients was observed when the GP was qualified in NP and the interaction between the qualification was also statistically significant (p<0.0001), but no independent effect of the qualification in NP was observed. See S3 Table for the identified determinants of hospitalization rates.

## Discussion

This study is, to the authors’ knowledge, the first to investigate potential differences in practice patterns of GPs additionally qualified in naturopathy in the German healthcare system. Among the investigated outcomes, an independent effect of the qualification was only observed concerning the rate of phyto-pharmaceutical prescriptions which was constantly higher when the GP was qualified in naturopathy. We observed interaction effects between the qualification of the GP and the age group and the sex of the patient in case of prescription and referral rates. In case of hospitalization rates interaction effects between the qualification of the GP and the age group of the patient could be demonstrated. This suggests that differences in practice patterns vary with respect to certain subgroups of patients. However, the magnitude of the observed differences, despite being statistically significant (in a large sample of patients), appeared to be rather small and their clinical relevance may therefore questionable.

As outlined in the background until now there are only a few studies comparing practice patterns and health care costs with regard to additional qualifications of GPs in NP/CAM. In contrast to the findings of the studies conducted in Netherlands and in Switzerland [10–12] we did not find independent effects of the qualification on hospitalization rates, referral rates or prescription rates in general. This difference might be explained by different methodological approaches, but also might be “real” differences in the health care systems of the involved countries.

Assuming that the sample of GPs included in our study are representative for Germany these findings suggest that conventional and naturopathic GPs in Germany are comparable in terms of their practice patterns and patient populations except the rate of phyto-pharmaceutical prescriptions. However, important differences in the attitude of healthcare between conventional and CAM GPs may not be reflected by our indicators. Salutogenetic strategies such as supporting self-management and inner resources, recommendation of home remedies and extensive consultations need a lot of time which is not adequately mapped within the German remuneration system. Therefore, we might not find differences in structural outcomes as used in our study (e.g. referral, hospitalization) but in patient relevant outcomes. The study of Studer points in this direction finding higher patient-reported quality of care in GPs with CAM qualification [12]. The perceived quality of care might be a reason that patients wish a better integration of CAM in general practice [20]. In an own qualitative study we have shown this for herbal medicine [21].

As in the German health care system there is no registration model for primary care a patient can each choose his or her GP. Therefore, it is important to differentiate selection effects on the basis of free doctors choice from "real" differences in quality of care as claimed by some authors of previous studies [10, 11].

### Strengths and limitations

A strength of our study besides the large sample size of patients is the robust methodological approach considering interaction effects and non-linearity in age-differences. A limitation of our study could be seen in the fact that we explored only general categories of practice patterns (prescriptions, referrals, hospitalizations). Those general categories are merely quantitative measures of practice patterns but do not give any indication for the quality of care. Furthermore, we included only a very limited number of covariates without considering morbidity. Even, if we know from preceding analyses morbidity levels across the practices are expected to be approximately similar [17], our study population sample cannot be assumed to be representative for Germany. Further studies are about to be designed on the basis of a larger sample in terms of GPs and patients.

These studies will include specific indications/diagnosis groups. Furthermore, causal interpretation would be facilitated by a before -after design; e.g. an investigation of practice patterns before and after the training in naturopathy.

## Conclusion

This study suggests that the rate of phyto-pharmaceutical prescriptions is generally higher when the GPs has an additional qualification in NP. Further differences are rather small without clinical relevance and may be subject to certain age groups and sex. Our findings build a robust basis for further analyses exploring CAM/naturopathy as part of primary health care.

## Supporting Information

S1 Data Study Data (data.sas7bdat). (SAS7BDAT)

S1 Table Determinants of prescription rates. (DOCX)

S2 Table Determinants of referral rates. (DOCX)

S3 Table Determinants of hospitalization rates. (DOCX)
