# Supplementary material for: Differences between Practice Patterns of Conventional and Naturopathic GPs in Germany
Source: PLoS One. 2016 Oct 3;11(10):e0163519. doi: 10.1371/journal.pone.0163519 (PMC5047621; doi:10.1371/journal.pone.0163519)
Supplement: S1 Table — (DOCX) [file pone.0163519.s002.docx]

S1 Table: Determinants of prescription rates

| **Type of prescription** | **Predictors** | **P value** |
| --- | --- | --- |
| Prescriptions overall | *Independent predictors* |  |
|  | Sex | 0,046 |
|  | Age group | <0.0001 |
|  | *Interactions* |  |
|  | NM × sex | <0.0001 |
|  | NM × age group | <0.0001 |
|  | Sex × age group | <0.0001 |
| Phyto-therapeutic prescriptions | *Independent predictors* |  |
|  | NM | 0,034 |
|  | Sex | 0,017 |
|  | Age group | <0.0001 |
|  | *Interactions* |  |
|  | NM × sex | 0,0003 |
|  | NM × age group | <0.0001 |
|  | Sex × age group | <0.0001 |
| Non-phytotherapeutic prescriptions | *Independent predictor* |  |
|  | Age group | <0.0001 |
|  | *Interactions* |  |
|  | NM × sex | <0.0001 |
|  | NM × age group | <0.0001 |
|  | Sex × age group | <0.0001 |

The individual model coefficients are omitted from this table for brevity and due to difficult interpretability in the presence of interactions. P values are based on Type III tests of fixed effects. Abbreviation: NM, naturopathic medicine.
