# Supplementary material for: Differences between Practice Patterns of Conventional and Naturopathic GPs in Germany
Source: PLoS One. 2016 Oct 3;11(10):e0163519. doi: 10.1371/journal.pone.0163519 (PMC5047621; doi:10.1371/journal.pone.0163519)
Supplement: S2 Table — (DOCX) [file pone.0163519.s003.docx]

S2 Table: Determinants of referral rates

| **Predictors** | **P value** |
| --- | --- |
| *Independent predictors* |  |
| Sex | <0.0001 |
| Age group | <0.0001 |
| *Interactions* |  |
| NM × sex | <0.0001 |
| NM × age group | <0.0001 |
| Sex × age group | <0.0001 |

The individual model coefficients are omitted from this table for brevity and due to difficult interpretability in the presence of interactions. P values are based on Type III tests of fixed effects. Abbreviation: NM, naturopathic medicine.
